# Bibliology; or Analytical Reviews of Medical Publications, Interwoven with Original Observations, and Practical Researches

**Published:** 1819-04-01

**Authors:** 


*Practical Observations on the Causes and Cure of Insanity.* By WILLIAM SAUNDERS HALLARAN, M. D. Physician to the Lunatic Asylum of Cork, &c. 1 vol. 8vo. pp. 213. Cork, 1818. (Second Edition enlarged).

OVER the link which binds mind to matter, a cloud—an almost impenetrable veil is spread. This cannot be denied, even by those heartless philosophers who look on mind, or what is termed *soul*, to be merely a *function* of organized matter; which, like that of sight or hearing, ceases to exist, when the organ itself is no longer capable of its office. Certainly the wreck of reason presents a most distressing picture of Human Nature! The loss of this most invaluable prerogative sinks proud man as far below the level of other animals, in respect to helplessness, as its possession raises him over them, in respect to power and intellectual cultivation. We are among those who believe that Insanity is a *corporeal* disease, while at the same time we abjure materialism. We believe that neither this nor any other morbid affliction was ever designed for us by NATURE; but that it, and all other diseases, result from a disobedience of, and aberration from her laws. They are punishments which we, in general, bring on ourselves, or are accidentally inflicted by others—but not, we trust, the dispensations of PROVIDENCE.

The brain, as the material organ through which the soul manifests its existence, may be deranged by a variety of causes, and in a variety of ways, so as to impede, or even totally prevent this intellectual manifestation, without any necessity for the annihilation of mind itself. In sleep, the soul shews itself not; yet it has not deserted its material fabric. In fevers, the *cerebral* functions are deranged, for a time, not the *mental* functions. When the fever is over, and the brain has regained its pristine state, the mental faculties are manifested as before.

On this point, then, we totally disagree with Dr. Hallaran, who argues for *disease* of the mind, while he denies the possibility of its *death.* He considers the *manifestations* as the *essence* of mind for the time being; and hence, says he,

> " If the manifestations of the mind be diseased, from any given cause, they naturally must betray an unsound mind, in the ordinary acceptation." 19.

To this we can by no means subscribe; but we shall not stop to argue on a point which, after all, can only be conjectural.

We agree with our author, however, that the *causes* of Insanity may be practically and usefully divided into mental and corporeal, while " the malady, though differing in its origin, is, in effect the same." The method of treatment, too, we know, must be modified by an attention to this important difference of cause—since *functional* disorder of the brain may often be removed by mere *moral* means, while *organic* derangements must have more *material* remedies.

1. *General and local Causes of Insanity.* Among these, our author justly places WAR, domestic and foreign. The unhappy political feuds and animosities which have so long distracted the sister isle, and, at one time, ripened into open rebellion, engendered, among other evils, a host of visionary views and consequent disappointments, followed by the pangs of suppressed licentiousness and arrogance. Others again, who, though " guiltless of their country's blood," beheld their property wasted, their friends destroyed, and the strongest ties of consanguinity broken, have sunk into hopeless apathy—a prey to wretchedness and despair. To these causes may be, in a great measure ascribed the augmentation of the insane lists within the last seventeen years in the asylums of Ireland.

From this dreadful scourge, the higher classes were not exempt. In some, it was the result of terror—ending in melancholia. In others, disappointed ambition was followed by gay, loquacious, and fanciful hallucinations. Both forms were equally incurable.

The abuse in spirituous liquors is another cause of Insanity in Ireland, of wide operation, the first and second attacks from which are generally within the reach of medical aid; but if the attacks are repeated, fatuity, epilepsy, or death is the termination.

*Religious dread* is a well known operative cause of Insanity; and it was, ten to one, more prevalent among Protestants than Catholics.

Respecting the *hereditary* nature of Insanity, we need not now raise a cloud of verbiage to puzzle common sense. Peculiarity of organization is transmitted from parent to progeny, and so is the disease dependent thereon. This is the plain meaning of the *mysticism* of susceptibility and predisposition, so long cherished by the disciples of John Hunter.

II. *Treatment.* Although it cannot be expected that Mania Furibunda will, in the first instance admit of a similar mode of cure to that which is the result of the depressing passions, producing melancholia, yet the ultimate treatment of each form of Insanity will not be found to differ very widely. The furious maniac is liable to be soothed, and finally benefited by moral means; while the melancholic patient often becomes violent and unmanageable, so as to require the intervention of evacuant medicines. So near, indeed, do the opposite forms of Insanity approach each other, that the one is frequently observed to *terminate* with symptoms which are peculiar to the other at the *commencement*; and vice versa. Still the impression of the original exciting cause supports pretty uniformly its genuine character throughout, and will not long bear a deviation from its appropriate specific treatment.

The first impression which the physician makes on the insane patient is of great importance, as it is generally either favourable or unfavourable. The attempt to argue him out of his hallucinated idea is worse than useless. The less notice, indeed, that is taken, even of his most obstinate phantasies, the less disposed will he be to retain them. On the contrary, it is better to coincide with his wildest extravagancies, unless where the delirium of fever may render strict silence necessary; or where the insane is disposed to commit an injury on himself or others.

In respect to the pathology of Insanity, our author is rather undecided; but he observes that—" in *all* instances, whether of the sanguineous or sanguineo-melancholic, he has long entertained the opinion that, in whatever degree the arterial action is exerted, there follows at the same time a torpor of the venous system, effecting a diminution of the equilibrium in the circulation, so essential to the preservation of health."

The observations of Dr. Armstrong, in this country, tend something to the same point. This last physician, whose accuracy of observation is well known, has seen several cases where mania was ushered in by the strongest signs of cerebral *congestion*, while the tone of the heart was extremely oppressed, the face very pale, the pupils dilated, the hepatic secretions disordered, and the skin cool and relaxed. In other instances, again, Dr. Armstrong has observed mania to commence with what is termed excessive determination of blood in the *arteries*, with swollen reddish face, ferretty eyes, full, bounding pulse, and preternatural heat of the surface, but especially of the forehead and hairy scalp. In the congestive variety the patients often complain, a little before the attack, of a load or confusion in the head, with an oppression about the heart, or at the epigastrium; while in the excitive variety, patients often complain of a pulsating pain or fullness in the head, the action of the heart being increased both in force and frequency. The congestive variety often passes into the excitive, as we see in many fevers.

From the pathological opinions of Dr. Hallaran, he has hitherto refrained from venesection, as much as possible—principally, however, from the circumstance of patients being received into public asyla in very advanced stages of the disease. In private practice, where, in some instances, he met with maniacal cases in the primary stages, he opened the temporal artery, which, by diminishing the excessive impetus of the heart, induced quiescence, and gave immediate relief. *Post mortem* researches did not appear to our author to afford satisfactory proofs of previously existing inflammation of the brain; on the contrary, he asserts, that the brains of those who had died under the direct influence of the insane orgasm, most generally exhibited decided evidence of relaxation, with a peculiar soft, white, flabby aspect of the entire substance, resembling more the appearance of curds than that solid, compact mass, which we observe to be the common consequences of inflammation. The ordinary course of protraction which Insanity is observed to run, militates, Dr. H. thinks, against the idea of inflammation of the brain as a cause of Insanity. And he adduces a curious instance of a young woman in the asylum over which he presides, where the paroxysms are furious to excess; her countenance exhibiting invariably a most terrific glare, and her uproar being hideous. The pulse, during the paroxysms, continue at about 96, full and hard; the skin hot and red throughout; muscular motion incessant and uncontrolable. To take blood, under these circumstances, is altogether impossible.

During the intervals, she appears as completely free from uneasiness, both of body and mind, as the most healthy person. In this case, our author asks, how it comes to pass, that if any thing like inflammation be present in the paroxysms, it should leave no trace behind it in the intervals? We answer, that inflammation and temporary local turgescence are very different things. A man may have apoplexy, the abolition complete of sense, from local engorgement of the cerebral vessels, but without inflammation. We are not, therefore, advocates for the use of *general* bleeding in these cases, unless the general excitement run particularly high; and this leads us to

1. *Local Bleeding.* In a great variety of cases of Insanity, especially in young subjects, the sense of heat throughout the scalp; the tinnitus aurium; the idea of loud voices heard from afar; and the coldness of the feet, evince the determination of blood to the head—or rather, congestion in the venous system of that organ. Apoplexy not unfrequently grows out of such appalling circumstances. Here there is no inflammation, yet the application of leeches is of the first consequence. They should be repeated as long as the superficial heat shall continue. Cupping glasses may be applied over the bites, in order to abstract a sufficient quantity of blood—all this may be done without making any sensible impression on the general circulation, yet with great advantage to the local congestion.

2. *Emetics.* Dr. H. has found that insane patients resist the operation of emetics, requiring triple the dose for people in health. He cautions against the full action of vomiting, "when the danger of over distension is to be apprehended" in the cerebral vessels. We have long considered this dread of distention in the head, during the operation of emetics, as truly chimerical. We know that there is not a more powerful mean of relieving local internal congestion, whether of the head, lungs, or abdominal viscera, than nausea and vomiting. This last drives the volume of blood to the surface with such force and rapidity, that all turgescences of the interior are instantly resolved. In saying this, we are not advocating the practice of emetics in mania, but stating a fact which may be applicable on many other occasions, and which is not sufficiently known or believed. Dr. H. recommends combinations of purgatives and emetic tartar, so as to excite nausea, and at the same time act on the intestinal canal. It is needless to say that these remedies are more peculiarly applicable to acute Insanity. Where, from turbulency, blood cannot be taken, antimony in proper doses, at moderate intervals, seldom fails to reduce the most stubborn maniac to a state of relative quiescence; and sleep very frequently follows with those happy consequences which succeed to a relaxation from inexpressible misery.

3. *Purgatives.* These are almost always of primary importance during the progress of an insane paroxysm. At the conclusion of fever, and even at the more advanced period of convalescence from mania, purging is invariably indispensable. The biliary secretion is very generally deranged in this disease; and therefore, a combination of calomel, jalap, and antimonial powder, in such proportions as shall secure them a direct passage through the bowels, will be very proper. After purgatives, a degree of reaction occasionally succeeds, marked by obstinate constipation, acute pains darting through the head, and complete sleeplessness. Under these circumstances, antimonials, singly or combined with neutral salts, are eminently serviceable. The indication for a continued use of the purgative plan will be drawn from the appearance of the stools. So long as they shall consist of dark matters, accompanied with scybala, and but slightly tinged with bile, the purgatives are to be continued.

4. *The circulating swing* was first proposed by Dr. Darwin, and brought into practice by Dr. Cox. Dr. Hallaran has found it very serviceable in those who were recently attacked, and after they had been sufficiently evacuated by purgative medicines. It was also useful in those inflexible maniacs, on whom no influence could be exerted in the administration of their medicines. Where the object is to produce free evacuations, the swing seldom fails on increasing its velocity to the degree required, gradually rather than by giving it rapidity at the beginning. In this way the stomach, bowels, and urinary passages, may be acted on in quick succession, particularly by reversing the motion of the swing every six or eight minutes, pausing occasionally, and stopping its circulation as suddenly as possible. The discharges which succeed are often surprising, both by their extraordinary magnitude and fœtor.

The circular swing, however, is not to be used indiscriminately, and not till after other remedies are employed. In what is termed intermitting Insanity, Dr. H. has found the swing peculiarly applicable, except where there is much determination to the head.

5. *Digitalis*, according to our author, is not eligible in any case, unless where the system is previously reduced by proper evacuants; and, in fact, its sedative properties cannot be usefully exerted, under the pressure of high arterial action. Dr. Hallaran, indeed, comes to similar conclusions with Dr. Sanders of Edinburgh, on the subject of digitalis; namely, that its *first* operation is stimulant.

"Its stimulant principle is unquestionably previously exerted; and by this principle does it seem chiefly to impart its benefits. The *sedative* quality of digitalis is secondary; it cannot therefore, with safety, be administered beyond a certain extent, any more than opium—to which, in its ultimate effects, it is greatly similar."

Dr. H. on this principle, prescribes digitalis with the greatest confidence of success.

"Digitalis has shewn its capability of restraining within due bounds, the action of the heart and arteries—so has it, in a remarkable manner, evinced its anodyne and soporific qualities."

Its exhibition must be watched. On the first appearance of pallor, nausea, vertigo, dilated pupils, slow intermitting pulse, with cold extremities, the digitalis should be laid aside, and the volatile alkali, and other cordials administered. It is principally, however, in those cases where carried to a large amount, that the remedy manifests the most decided and the most permanent proofs of superiority.

6. *Opium* appears to be a very doubtful, or rather dangerous remedy in Mania, where there is any degree of arterial excitement; and of late years, it has been seldom used in the Cork Lunatic Asylum, having been superseded by the swing and digitalis. It may still, however, be a question, whether, in the generality of instances, a full and timely dose of opium, by interrupting the quick succession of morbid sensations, might not, in the event of its operation, so fully tend to dissever the catenation as to make way for the gradual return of rational perceptions: but it is a remedy which cannot be persisted in longer than the first effort; especially where the occasional causes are such as induce an over distention of the cerebral vessels, and a febrile diathesis.

7. *Camphor* has been loudly extolled in Mania; but our author has found it uncertain and inefficient.

8. *Blisters* have been too indiscriminately applied, especially to the head. In the more advanced stage of Insanity, or rather towards the decline of the paroxysm, where a want of energy, and an incapacity to participate in the usual objects of volition succeed to the previous temper of activity, the occasional repetition of a blister round the lower part of the neck is often found beneficial. Their efficacy may, in certain cases, be more complete, by placing them low down, as on the calves of the legs.

9. *Mercury* is not generally applicable to the cure of Insanity, except where the causes have been retrocedent gout, or hepatic derangement.

"Although I cannot (says Dr. H.) decide favourably on the specific properties of mercury in this disease, I am far from limiting them to its purgative quality:—the equable and general stimulus which it affords to the system at large, by an evident action on the absorbents, has taught me to entertain a high opinion of its utility as a preparative for the digitalis."

10. Calomel, as answering a two-fold purpose, of freeing the bowels and acting on the general system, is, of all other preparations, the best. The combination of calomel and tartarized antimony, in the early stages of the complaint, where the influence of antimonials was desired, invariably promoted the evacuant qualities of the latter — in other cases, it may be combined with antimonial powder.

We may here state the sentiments of Dr. Armstrong on this point; sentiments which accord pretty uniformly with the results of our own experience. In the first two weeks of attack, Dr. A. generally bled, both from the arm and temporal artery, until the cerebral turgescence was relieved, and the action of the heart restored to a more natural state, evacuating the bowels, at the same time, by calomel, jalap, and salts—the calomel being given in full doses, so as to procure both its constitutional and purgative operation. After that period, he drew blood by leeches or cupping about twice a week, ordering a saline purgative every second morning, and giving calomel daily, in such doses as to insure a moderate but constant ptyalism for some time: two thirds of the *recent cases* of madness recovered within the first three or four months, under this treatment; though hardly any of the patients shewed signs of convalescence until the mouth had been affected for some weeks by the mercury, and until some degree of emaciation took place. When, as is generally the case, we find a difficulty in impregnating the system with mercury, this is an indication that evacuations are required, which always tend to render the system pervious to the influence of mercury. When ptyalism is once induced in mania, it is easily kept up by moderate doses of calomel or blue pill, or by mild mercurial frictions. After sufficient evacuations, he has often combined small doses of opium with the alteratives; and whenever there was much nervous irritation, this combination always appeared more or less beneficial.
*On Typhus, p.* 304, *Second Edit.*

11. *Warm and Cold Bath.* In the early stage of Mania, and where the necessity for active evacuants still continues, no greater degree of heat than the temperature of the body, at the time being, can be unproductive of mischief.— In fact, the warm bath, at any temperature, is altogether unsafe in the first stage of mania; neither does it seem very admissible in the second or improving stage; in short, where stimuli are improper, the warm bath is inadmissible.

In the third, or convalescent stage of mania, the warm bath, in Dr. Hallaran's opinion, is highly beneficial. The secretory organs are thereby excited into healthful action; especially the liver, as evinced by a critical flow of yellow bile from the intestines; a general forerunner of complete recovery.

We should begin with a temperature of 96°, at the highest; and the continuance in it must be regulated by the immediate state and strength of the patient. To obviate *deliquium animi,* napkins wrung out of cold vinegar and water, and applied entirely round the head, in the form of a turban, and frequently renewed while in the bath, will be serviceable. This is something of the same plan as that of Messrs. Delahoyd and Lucette, which made so much noise a few years ago. Moderate exercise in a dry, open atmosphere, immediately after coming out of the bath, ought, in general, to be enjoined.

12. *Shower Bath.* Here Dr. Hallaran conceives, that the rules laid down by the late Dr. Currie, in respect to cold affusion in fever, will generally apply in mania. We must remember, however, as is well observed by a late excellent writer, that much harm may be done by persevering in the cold shower bath, after the tone of the heart and arteries has been subdued; for it then may increase or occasion fullness in the vessels of the brain, confirm the insanity, or induce an attack of apoplexy, palsy, or epilepsy. Where there are determinations to the head, we have found more permanent advantage from wetted cloths to the scalp, as beforementioned, keeping the head high, in order to retard the tide of arterial blood to the head, and accelerate its return by the veins; a circumstance of great importance in all severe affections of the cerebrum. In those cases, as Dr. Armstrong observes, which are strictly congestive, the cold affusions cannot be safely employed; but the *warm* shower or slipper bath may be beneficial, in combination with the depletory practice. Where the mercurial course, with the means before enumerated, had failed to remove all the sypmtoms of insanity, Dr. A. has generally persevered in the use of the warm or cold shower bath, with tolerably active purgatives; and has not often been disappointed, where the disease was not of long standing.

13. *Seclusion from Light.* The stimulus of light on the optic nerves, and through them on the brain, is exceedingly instant in its effects, and injurious in its consequences, in insanity, especially of the acute kind. Maniacs, however, do not commonly complain of the effects of light on their senses; on the contrary, they rather court it. The regulation of light then, in the apartments of the insane, is of the utmost consequence. Total darkness, indeed, is not to be preferred. A moderate and equable degree of light is at all times necessary, so that surrounding objects may be distinctly viewed, without the danger of deception by optical deformities. The light should be received into the room of an insane patient indirectly, or from a northern aspect; it ought also to be admitted from the highest elevation which the room will afford, so as to avoid all intercourse from without, and prevent irritation, even from inanimate objects. Persons far advanced in convalescence have often been thrown suddenly back from their inability to bear the impulses of light, or the impressions and associations resulting from officious intrusions. It is, therefore, of great importance "that no objects be left in the way of observation, which may revive former associations, either of *a grateful or offensive nature.*" In convalescence from this disease, rural scenery has always been found to have a more tranquillizing effect than the scenes of busy life, whence a quick succession of ideas is forced upon the imagination, and that with serious mischief.

14. *Exercise* forms a material department of regimen in the convalescent state. The extreme degree of muscular debility which invariably succeeds a maniacal paroxysm often degenerates into such a rigidity of fibre, as tends even to a curvature of the spine, and inability to extend the lower extremities. In pursuing this measure, the exercise and business of horticulture seems to merit the most decided preference. Sedentary employments, of all descriptions, are ill calculated to the dignity of the imaginary STATESMAN OR THE MIGHTY POTENTATE! and equally so to the convalescent maniac, whose chief happiness consists in the free exercise of his faculties in the open air.

15. *Diet.* This must, in some measure, be regulated by the previous habits of the patient; but, upon the whole, the farinacea are preferable to the animal dishes. In the Lunatic Asylum at Cork, there are certain seasons of the year, when allowance of animal food is made to the insane patients. The consequences, however, on these occasions, are uniformly the same—a scene of uproar. Animal food therefore tends, generally speaking, to the aggravation of insanity; especially where there is a prevalence of those acute appearances which denote the insane orgasm. In the more advanced period of convalescence, and under the symptoms of debility, owing either to age, or to the protraction of a paroxysm, a gradual indulgence in animal food may be allowed. Wine and fermented liquors are, of course, equally injurious, unless in the most urgent cases. The insatiable cravings for food or drink, which maniacs frequently discover, are never to be considered as demanding the same indulgence as the natural inclination which denotes the tendency to real amendment.

CHRONIC INSANITY.

This is what succeeds the acute form where recovery does not take place. This form composes the great majority of cases confided to the care of the private practitioner. Its treatment bears, in the generality of instances, a strong reference to that pursued in the primary state—varying very little more than in degree, and requiring to be regulated in strict conformity with the force and continuance of the paroxysms.

We are disposed to agree with Dr. Armstrong in dividing this form into two varieties—the one arising from a strictly *congestive* state of the brain, the other connected with arterial excitement. The congestive form is preceded by paleness of the face and skin; watchfulness and restlessness; uneasiness in the head; occasional load at the heart, and derangement of the hepatic function. The pulse is almost always weak or oppressed during the day, the surface being damp with cold perspiration. A slight febricula and imperfect reaction take place towards evening. The other form connected with arterial excitement is generally preceded by disturbed sleep; head-ache; quickness and firmness in the pulse; white tongue, and increase of heat at nights. In this, as in the other form of chronic mania the liver is very apt to be diseased, either primarily or secondarily.

Where chronic insanity has decidedly assumed the periodical type—the recovery may be greatly promoted by the cautious introduction of the liq. arseniatis potassæ.

*Seclusion and Separation from Friends.* Universal experience has decided on the propriety of early removal and non-intercourse of the insane with their intimate friends and relatives. Yet it is to be lamented (however amiable the failing) that when this afflictive misfortune comes home to our own doors, our sentiments and opinions are re-modified, and reason is blinded by sympathy! The tenderest emotions of friendship and affection become strongly engaged. Doubts arise, and objections are urged by those whose bounden duty it is to weigh well the nature of the sacrifice to which they are called upon to submit. They ought to bear in mind, that the assiduities and attention of relatives are never received by the insane as proofs of friendship or kindness; and, that their ultimate effect is rather to irritate than soothe. With the insane, the dearest objects of affection become exposed to the most invincible hatred. If that degree of restraint and privation, which is necessary for the welfare of the insane, be imposed while intercourse with relations exists, then will a constant source of irritation be generated, since the patient will be thoroughly convinced, that this coercion and this privation is with the consent at least, if not by the express desire of the said relatives. Hence they are incensed at such cruel conduct, and have often been known to retain a lasting hatred against their nearest kindred, even when they had recovered from the hallucination; for it must not be supposed that the insane are ever unmindful of what passes around them.

The interference of domestics, in the periods of violence, is sure to create an additional source of irritation. Any assumption of authority on their part, conveys the idea of an inverted order of things, to which a feeling of resentment never fails to succeed. Servants too, when incautiously confided in, will frequently from selfish policy, defeat the views of the physician, by yielding to all the dangerous propensities of the patient, or by insidiously condemning the means adopted for his relief. Their inexperience and irresolution, even with the best intentions, set order at defiance, and the apartment of the sick, through want of method, becomes repeatedly the scene of tumult and confusion. In short, half-measures, in the treatment of the insane, with the appearance of decision, are even worse than total inactivity: consistency throughout is indispensible. Seclusion from friends, from home, and from domestics is absolutely necessary.

Dr. Hallaran's work, though somewhat too *popular* in its construction, is certainly respectable; and indicates a mind fraught with practical knowledge, and strongly embued with the feelings and the principles of humanity.

---

### Surgery.

### VI.

*Pathological and Surgical Observations on Diseases of the Joints.* By B. C. BRODIE, F. R. S. Assistant Surgeon to St. George's Hospital, and Lecturer on the Theory and Practice of Surgery. One Vol. 8vo. pp. 329. Six Plates. 1818.

MR. BRODIE, as our readers well know, has long cultivated this branch of pathological surgery with great success. The talents which he possesses, the opportunities he enjoys, and the zeal he displays, all conspire to raise expectation, whenever a work is announced from his pen. To say that these expectations will not be disappointed, is feeble praise; but to give a copious analysis of the work itself, will assuredly be the most effectual mode of paying due homage to its intrinsic merits, and of diffusing its valuable contents through a wider circle than the volume itself might probably traverse.

The pathology of the joints, particularly in respect to diagnosis, has been hitherto involved in much obscurity, producing a corresponding confusion in the application of remedies. Although in the ulterior stages of articular disorganizations, every tissue and structure may be expected to suffer; yet there is good reason to believe, that the morbid action begins in some *one* particular texture at first; and that the nature and treatment of the disease is *then* modified considerably, according to the mechanical organization, or the vital properties of the part in which the disease originates. It is true that, in this volume will be found the substance of Mr. Brodie's papers on the same subject, in the 4th, 5th, and 6th volumes of the Medico-Chirurgical Transactions, but there will also be found much original matter, and a confirmation of our author's preceding observations.

### CHAP. I. *Inflammation of the synovial Membrane of the Joints.*

Mr. Brodie remarks, that hydrops articuli may sometimes arise from diminished action of the absorbents, or increased secretion. We are strongly disposed to believe that, in every instance, it is the product of inflammation, chronic or acute.

*Case* 1. A middle aged man was admitted into St. George's Hospital. The knee joint was swollen and painful, with stiffness and fluid in its cavity, the swelling extending some way up the anterior part of the thigh. It subsided under the use of blisters and liniments. Two months afterwards, he was seized with another disease and died. On dissection the synovial membrane was found much diseased, and more capacious than natural, extending up the anterior surface of the femur, at least an inch and a half higher than usual. Its whole internal surface was of a dark red colour, except where it covered the cartilage; the vessels being distended as in ophthalmic inflammation. A thin flake of coagulable lymph was effused.

This and many other cases authorise the conclusion that synovial inflammation occasions, 1st. a preternatural secretion of synovia; 2d. effusion of coagulable lymph; 3dly, in some cases a thickening of the membrane—a conversion of it into a gristly substance.

*Etiology and Symptomatology.* The disease is most frequent in adult age, which is the reverse of some articular diseases. It may be an extension of rheumatism; the effect of too great mercurialization, &c. in which cases, it is generally mild. It sometimes attacks all the joints at once; sometimes in succession; sometimes locally. The application of cold is, upon the whole, the most frequent source of the complaint. Hence its frequency in the knee-joint may be accounted for, where the articulation is so thinly covered. Although in itself a severe disease it is often confounded under the alarming name of white swelling, with other diseases which are still more serious.

This inflammation is generally chronic in its form, which, while it injures, does not entirely destroy the functions of the joint. If not treated properly and early, it may harrass the patient, as in ophthalmia, for weeks, months, or even years.

Pain is the first symptom—generally confined to a part, but sometimes universal over the joint. It increases during the first eight or ten days, when it is at its height. Swelling begins in a few days after the pain, evidently from fluid in the joint, which may be felt to undulate at first, before the membrane is morbidly thickened, when the swelling then consists more of solid substance, and the natural mobility of the joint is in a greater degree impaired. The form of the swelling is not that of the articulating ends of the bones, but depends on the situation of the ligaments and tendons, which resist it in certain directions and allow it to take place in others. The hip and shoulder are less frequently the seat of the disease than the superficial joints; in the latter the effused fluid can be felt to undulate, the swelling being perceptible through the muscles. When the hip is affected in the first instance, a tumefaction may be observed in the groin, and in the nates also, but where the disease has existed for some time, the nates assume a flattened appearance, in consequence of the glutei muscles becoming wasted from want of use.

After synovial inflammation has subsided, the fluid is absorbed, and, in many instances, the joints regain their natural figure and mobility; but in the majority of cases, the stiffness and swelling remain, and then the patient is very liable to relapse.

Mr. Brodie very justly observes, that the boundaries of acute and chronic inflammation do not admit of being well defined. These terms very sufficiently express the two extremes, but not the numerous intermediate degrees of inflammation.

*Treatment.* Where the disease occurs in consequence of irregular or ill-conducted courses of mercury, sarsaparilla is useful. When from rheumatism, opium with diaphoretics, or the infus. colchici autumnale may be employed. In some other instances, when several joints have been affected at the same time, Mr. Brodie has employed, with benefit, moderate doses of some mercurial preparation.

Topical remedies, however, are, upon the whole, of more importance than any other; and of these we shall proceed to speak.

In the acute form, leeches may be applied to the neighbourhood of the part, and if there be much symptomatic fever, blood may be taken from the arm, and repeated pro re nata, attention being paid to the state of the bowels in the mean time. If the swelling and tension of the parts be considerable, the pain will be best relieved by warm fomentations and poultices; otherwise, cold evaporating lotions produce a better effect. Under this treatment, the acute inflammation soon subsides.

The chronic inflammation is relieved more slowly. In the first instance the joint should be kept perfectly quiet; leeches or cupping, especially the latter, repeated twice, thrice, or oftener; and in the intervals, compresses are to be laid on the part, moistened with some cold lotion. When the violence of the inflammation is subdued, a blister may be applied, or rather a series of blisters, which Mr. Brodie thinks is better than one blister kept open with savine cerate. If the synovial membrane of the hip be inflamed, the blisters may be applied to the groin and nates, but if the disease be in the wrist, it may be applied to the lower part of the fore-arm.

Under this treatment the tumour will greatly subside, even if it be solid, and composed of coagulable lymph. When the inflammation is in a great measure subsided, a moderate degree of exercise of the joint is beneficial; liniments, such as the liniment. camph. compos. made stronger by the addition of liq. ammoniæ and tinct. lyttæ, or ol. terebinth. may be used; or the following:—℞. Olei olivæ ℥iß; acidi sulph. ʒß. M. fiat linimentum. This, for common working people, will not be too strong; but for those in higher life, whose cuticle is thinner, it may be made with a larger proportion of the oil. The tartar emetic ointment also is very useful. No other remedies seem to be productive of much benefit.

"Issues and setons are of no service, except where there is reason to believe that a secondary disease has begun to take place, in the form of ulceration of the cartilages."

For the swelling and stiffness remaining after inflammation, free exercise and friction, with mercurial ointment and camphor may be cautiously used.

Chap. II. *Ulceration of the Synovial Membrane.*

When an abscess takes place in a joint, the matter is discharged through an ulcerated opening in the synovial membrane. The most remarkable circumstance in such cases is, that a disease apparently slight, and of a part which is in no way concerned in the vital functions, should produce such a degree of disturbance of the constitution as to occasion death. This, however, is not a solitary circumstance, as we know that in many other instances an impression made upon a small part of the nervous system, may derange, and ultimately destroy the functions of the whole animal machine.

### Chap. III. *Morbid Change of the Structure of the Synovial Membrane.*

This change is peculiar to the membranes in question, as our author has never seen an instance of the kind in the serous membranes—this is ulceration. The following case, condensed from Mr. Brodie's work, will illustrate this subject.

Martha Manners, ætat. 26, was admitted in 1813, into St. George's Hospital, on account of a disease in her right knee. In June, 1811, her knee began to swell and become stiff, which increased, though slowly. On admission into the hospital, the knee measured two inches in circumference more than the other; swelling elastic; prominent at the upper and lower part of the joint, not having the form of the articulating ends of the bones. The joint admitted of motion, but the leg could not be completely bent or extended on the thigh. All remedies proved fruitless. An abscess presented itself by the side of the ligamentum patellæ, and was opened. Pain continued; the general health suffered, and the limb was ultimately removed. On examining the joint, an ounce of matter was found in its cavity. The ligaments were found in a natural state. The synovial membrane, where it formed the loose folds, extending from one bone to the other, and also where it was reflected over the bones themselves, the crucial ligaments, and the fatty substance of the joints, had completely lost its natural appearance. It was converted into a pulpy substance, in most parts about a quarter, but in some parts nearly half an inch in thickness, of a light brown colour, intersected by white membranous lines, and with red spots formed by small vessels, injected with their own blood-vessels. The synovial membrane on the edge of the cartilaginous surfaces had undergone a similar change of structure, but only for a small extent. The semi-lunar cartilages were entire, but in a great measure concealed by the pulpy substance projecting over them. The cartilages covering the bones, in a few places, were in a state of incipient ulceration.

Our author here introduces several examples of the same disease, in different stages of its progress. The morbid action evidently originates in the synovial membrane, which loses its natural organization, and becomes converted into a thick pulpy substance, of a light brown colour, sometimes reddish, intersected by white membranous lines. As the disease advances, it involves all the parts of which the joint is composed, producing ulceration of the cartilages, caries of the joints, wasting of the ligaments, and abscesses in different places.

This disease our author believes to belong to the same order with tubercles in the lungs, scirrhus in the breast, fungus hæmatodes of the testicle, and to these also it bears a near resemblance in its progress. In every case which he has seen, it has ultimately terminated in the destruction of the joint. It is rarely met with except in the knee, and this may be owing to the influence of the external cold.

*Symptomatology.* In the origin of the disease, which seldom occurs after puberty, there is a slight degree of stiffness and tumefaction, without pain, and producing only the most trifling inconvenience. In the end, the joint loses its capacity of motion; the swelling is soft and elastic, and gives to the hand a sensation as if it contained fluid. If one hand only be employed in the examination, the surgeon may be deceived, and believe that there is a fluid; but the absence of fluctuation, when both hands are employed, detects the complaint. The patient experiences little or no pain until abscesses begin to form, and the cartilages to ulcerate. At this period the patient becomes affected with hectic fever—loses his flesh, and gradually sinks unless the limb be removed. In general, one or two years elapse before the disease reaches its most advanced stage; and sometimes the period is much longer.

The *diagnosis* is not very difficult. The gradual progress of the enlargement, and stiffness of the joint without pain, and the soft elastic swelling without fluctuation, enable us to distinguish it readily from all other morbid affections to which the joints are liable.

*Treatment.* When a part is swollen and rigid in consequence of inflammation, the swelling and rigidity may be often dispersed; but when an organ has completely lost its natural structure, it never can be restored. The disease of the synovial membrane under consideration is equally incurable as tubercles of the lungs, cancer, &c. By means of rest and cold lotions, the progress of the disease may be somewhat checked, as the suppuration of tuberculated lungs may be retarded by occasional bleeding, and a mild climate. Where there is considerable pain in consequence of the cartilages beginning to ulcerate, some benefit may be derived from warm fomentations and poultices; but still these will only check the progress of the disease—amputation is the final cure.

### Chap. IV. *Ulceration of the Cartilages of the Joints.*

The articular cartilages in the adult possess no vessels capable of carrying red blood—inflammation is not in them a frequent occurrence; when it does take place, it terminates in ulceration, and not in the formation of bone. This ulceration may be the consequence of inflammation of the cartilage itself, or of the bony surface to which it is connected; but in many instances, there are no evident marks of inflammatory action having preceded it, either in one part or the other. Ulceration in the articular cartilages is not attended, as in that of soft parts, with the secretion of pus — this is worthy of being attended to. When cartilaginous ulceration takes place in the superficial joints, it constitutes what has been termed white swelling. In the hip, it has been termed morbus coxarius, scrofulous hip, &c.

*Case.* A boy was brought to St. George's Hospital, in April 1810, on account of a disease of the left hip. "The nates were wasted and flattened; there was pain in the hip and knee; and a large abscess had formed, which produced a tumour on the outside of the thigh. An issue was made with caustic behind the great trochanter. About a month after his admission, the skin over the abscess having become inflamed, I made an opening in it with a lancet, and half a pint of pus was evacuated. The orifice made by the lancet, healed by the first intention, but in a few days, pus was again collected in the abscess, and the tumour was larger and more tense than ever. The limb became shortened, the abscess burst externally, the boy became affected with hectic symptoms, and died on the 21st of October. On examining the body, the abscess was found communicating with the left hip. The capsular ligament and synovial membrane were not distinguished from the other soft parts, forming the parietes of the abscess. There was no vestige of the round ligament, and no remains of cartilage on either of the bones composing the joint. The head of the femur was reduced by caries, to about one half of its natural size; and from the same cause, the acetabulum was rendered deeper and wider than is natural. At the posterior part, the margin of the acetabulum was more extensively absorbed, and the head of the femur had been drawn out of its cavity, and was lodged on the dorsum of the ilium. No other disease had been suspected to exist during life. If the boy had ever complained of pain in the right hip, the circumstance had been overlooked, on account of the greater disease in that of the opposite side. Having accidentally cut into the joint of the right hip, I found the cartilage covering the head of the femur, absorbed for about one third of its extent, and the surface of the bone, which was in consequence exposed, was covered by a thin layer of coagulable lymph. The cartilage lining the acetabulum, and all the soft parts belonging to the joint, were in a perfectly natural state, and the bones were of the ordinary texture and hardness."

From many cases here related, and observed in practice, our author is led to the following conclusions:—1. That in the advanced stages of the disease, all the parts entering into the composition of the joint become deranged in structure. The soft parts are blended into a confused mass. Sometimes the head of the femur is completely destroyed, the neck only remaining; or the projecting margin of the acetabulum is entirely absorbed, leaving a broad carious surface of the os innominatum.—2d. At all periods of the disease, the cartilages are found in a state of ulceration, the surrounding soft parts not being materially affected, except in advanced states of the disease. From these circumstances, our author concludes, that in the ordinary cases of caries of the hip, the cartilage is the part primarily diseased. These ulcerations extend to the bones, and then abscesses form in the joint, which afterwards make their way by ulceration, through the synovial membrane and capsular ligament into the thigh, or nates, or even through the bottom of the acetabulum, into the pelvis or rectum. After things come to this pitch, the synovial membrane and capsular ligament become inflamed and thickened; the muscles are altered in structure; sinuses are formed in various parts; and at last all the soft parts are blended together into one confused mass, resembling the parietes of an ordinary abscess.

In some cases, the ulceration of the cartilage of a joint begins on that surface which is connected to the bone. Mr. Brodie found that, in many instances, previously to ulceration, the cartilage undergoes a remarkable change of texture, becoming soft, and assuming a fibrous appearance, all showing that cartilage, as well as other parts, is capable of ulcerating from the action of its own vessels.

*Symptomatology*. The articulo-cartilaginous ulceration occurs at any period of life, but principally in children, or in adults under the middle age. The hip is the joint most liable to this disease, as the knee is to synovial inflammation. Sometimes the patient can trace his complaint to a local injury; often no cause can be assigned. When in the hip, pain, and a slight degree of lameness are the only symptoms at first. The pain at first resembles that of rheumatism, being referrible to various parts of the limb in different individuals. As the disease advances, the pain becomes exceedingly severe, particularly at night, when the patient is continually roused from his sleep by painful startings of the limbs. In most instances, the pain is referred to the hip and knee also, being most severe in the latter; sometimes there is pain in the knee and none in the hip. The pain is always aggravated by motion of the joint, and still more by pressure of the ulcerated cartilaginous surfaces against each other. Hence the patient is unable to support the weight of the body on the affected limb; and if the patient be placed on an even surface, in an horizontal position, and the hand of the surgeon be applied to the heel, so as to press the head of the femur against the concavity of the acetabulum, violent pain is the consequence. This circumstance is well deserving of attention; and no one should attempt to give an opinion as to the nature of the disease connected with the hip, without having made an examination in the manner described.

Soon after the commencement of the complaint, the hip-joint will feel tender on pressure, either behind or before. The absorbent glands become enlarged, with some slight tumefaction in the groin. It is curious, that in some cases there is *tenderness* of those parts, as the knee or leg, where the sympathetic pain is complained of. This is conformable to what we see in other morbid sympathies. Thus the passage of a calculus down the ureter will sometimes occasion pain, tenderness, swelling, and even smart inflammation of the testicle.

In the progress of the disease, the nates lose their usual convexity, and present a flattened surface. In the early stage of the disease, the patient complains of the affected limb being longer than the sound one; but this is only an apparent, and not a real elongation, which may be ascertained by placing the patient in the horizontal position, so that both thighs make the same angle with the pelvis, when, if the distance from the spine of the ilium to the patella be measured, no difference will be perceptible.

"The apparent elongation is produced by the position of the pelvis being altered, in such a way that the crista of one ilium is visibly depressed below the level of that of the other. P. 146.

The shortening of the limb, which takes place in the advanced stage of the disease is usually, but not always, the precursor of the abscess. The formation of matter is also indicated by an aggravation of the pain, by more frequent spasms of the muscles, by a greater wasting of the whole limb, and by the circumstance of the thigh being bent forward, and being incapable of extension, without such an increase of the patient's sufferings as he will be unable to endure. At the same time the pulse becomes quick, the tongue furred, and the whole system is in a state of preternatural excitement. The abscess usually shews itself in the form of a large tumour over the vastus externus muscle—sometimes on the inside of the thigh, bursting and discharging a large quantity of thin pus; and in the worst cases a copious suppuration continues until the powers of the patient are exhausted, and he sinks in a state of emaciation, under the symptoms of hectic fever. Adults hardly ever recover from these states of the disease; but children sometimes, though with complete anchylosis of the joint.

When the cartilages of the *knee* are ulcerated, there is pain in the affected joint—at first slight, and relieved by rest; but, by degrees, becoming more severe and constant, especially at night. The pain is referred principally to the inside of the head of the tibia, and is aggravated by motion.

The ulceration of the cartilages of the knee differs with respect to its symptoms, from inflammation of the synovial membrane in this, that the pain in the former is slight in the beginning, and gradually becomes very intense; which is the very reverse of what happens in the latter. In ulceration of the cartilages also, the pain, in the first instance, is unattended by any evident swelling; which comes on, never in less than four or five weeks, and often not until several months have elapsed.

The swelling is usually trifling, appearing greater than it really is, in consequence of the wasting of the muscles of the limb. It has the form of the articulating ends of the bones—that is, the natural form of the joint. No fluctuation is perceptible, as where the synovial membrane is inflamed; nor is there the peculiar elasticity which exists where the synovial has undergone a morbid alteration of structure. But few cases occur in which the disease is attended with a collection of fluid in the joint; and in which, therefore, the tumour has a form different from that which has been described, and giving to the hand a distinct sense of fluctuation.

*Treatment.* Where there is ulceration of the cartilages of a joint, it is natural to suppose, that motion must be favourable to the progress of the disease.

Mr. Brodie has known some cases in which rest alone was sufficient to produce a cure. In all cases the symptoms of the disease are aggravated by exercise, and therefore perfect quietude is the most important circumstance to be attended to in the treatment.

The immediate relief, as Mr. Brodie observes, which sometimes follows the application of caustic to the skin, or the surface of an issue, when the limb is under precisely the same circumstances as before with respect to rest; and the return of the symptoms, which, in many instances, follows the early healing of an issue, are very difficultly accounted for, though they sufficiently prove the efficacy of the remedy.

Where the cartilages of joints are in a state of ulceration, *and in these cases only,* Mr. Brodie has found caustic issues useful. Setons and perpetual blisters operate in the same manner, and are proper under the same circumstances. The actual cautery is not recommended by our author. Where inflammation is brought on, with increase of pain, by too freely exercising the limb, then bleeding may be employed with advantage; but otherwise, this species of evacuation he has not found useful; indeed, no analogy would lead us to expect benefit from bleeding in ulceration. In the early stage, the warm bath is sometimes serviceable; plasters, liniments, and embrocations, are entirely inefficacious—friction invariably injurious.

Our author has shewn that articular cartilaginous *ulceration* may go on for a considerable time, without suppuration. This is of much practical importance to know, since the prospect of cure is much greater before than *after* an abscess has formed.

When the cartilages of the hip are ulcerated, the patient should be confined to a couch, or to his bed, and if the disease be far advanced, the limb should be supported by pillows and cushions properly disposed for favouring anchylosis, by preventing motion.

In young children, blisters afford complete relief, when applied round the nates, trochanter, and in the groin. A blister kept open by means of savine cerate, is more efficacious than repetitions of them. In children above the age of eight or ten years, and in adults, the same treatment is useful in the very early stage of the disease; but in the more advanced stage, issues made with caustic appear to be more efficacious than any other. The hollow behind the great trochanter of the femur is, in many respects, the most convenient situation for the application of the caustic; but in some cases, the application of it on the outside of the hip is attended with better effects. The skin of this part is, in fact, nearer to the joint than the skin behind. The skin in the groin is still nearer to the hip than on the outside; but the large vessels and nerves of the thigh forbid the use of the caustic at this part. A slough may be made with the *potassa fusa*, in the adult, half an inch in breadth and two inches in length, behind the great trochanter. If this fails in giving relief, a second slough of a smaller size may be made on the anterior edge of the tensor vaginæ femoris muscle, which will often afford relief, when the first has been inefficient. Mr. Brodie prefers keeping the issue open by simply rubbing the surface with the caustic potash, or with the sulphate of copper, twice or three times in the week. The pain produced by the caustic is very considerable, but the relief is in proportion. In general, there is some degree of abatement on the caustic being applied; but complete relief of the symptoms is not very common, immediately after making the issue. To relieve great pain, a local bleeding, or a blister may be serviceable; even a blister to the seat of the *sympathetic* pain will often relieve the real pain. Although caustic may be dangerous in the groin, there is not the same objection to a seton there. Mr. Brodie was led to adopt this treatment some years ago, partly from observing that the skin of the groin is nearer to the hip joint than the skin elsewhere, and partly from an expectation that the making a seton over the trunk of the anterior crural nerve might be particularly calculated to relieve the pain referred to those parts to which the branches of that nerve are distributed. The results of this practice more than realized his hopes. In many cases the seton occasioned very speedy relief of the pain; in other cases, however, it failed in producing the like good effects; but these cases have borne only a small proportion to those in which it has succeeded. On the whole, Mr. Brodie is led to conclude, that where the pain is very severe, the seton in the groin is more calculated to afford immediate relief than the caustic issue; but that it is not equally efficacious in checking the progress of the disease, as in lessening the violence of the symptoms, and that the caustic issue can be better depended on for the production of the cure. To make a seton in the groin, a curved seton needle is best. The seton may be introduced obliquely on the anterior part of the joint, including from an inch and a half to two inches of the integuments.

These observations are, most of them, applicable to ulceration of the cartilages in other joints. In all cases, a state of the most perfect quietude is indispensable. If in the lower extremity, the horizontal position should be enjoined; if in the upper, the arm should be in a sling. Where the knee or elbow is affected, the caustic or perpetual blister may be employed; in the knee, a narrow slough may be made on each side of the patella, by rubbing the skin with savine cerate—or a couple of caustic issues, one on the anterior, the other on the posterior part of the joint.

It is curious, that in some cases, although caustic issues and blisters give relief at the beginning, yet, after a certain time, they appear to do harm instead of good; and more relief is obtained by leaving them off.

When abscess in the joint occurs, Mr. Brodie has not found Mr. Abernethy's method of evacuating the matter, to be attended with any particular advantage, in case of carious joint. Neither emetics, electricity, nor pressure, produced any good effect after the matter had formed. The *early* opening of such abscesses is not recommended by our author. Rest, and the means already mentioned, should first be employed. In articular abscesses, the cavities are sinuous, and the matter is pressed out with difficulty—the pressure too is productive of bad consequences, as it causes the inner surface of the cysts to inflame.

"The practice which has appeared to me, on the whole, the best, is the following An opening having been made with an abscess lancet, the limb may be wrapped up in a flannel wrung out of hot water, and this may be continued as long as the matter continues to flow of itself. In general, when a certain quantity has escaped, the discharge ceases, the orifice heals, and the puncture may then be repeated some time afterwards; but where the puncture has not become closed, I have seldom found any ill consequences to arise from it remaining open." P. 187.

In young persons, even the smallest quantity of pus in a joint, in this disease, considerably diminishes, and in the adult very nearly precludes the possibility of cure, except by amputation. But if the surgeon sees the patient before matter has actually formed, then he may pretty confidently calculate on success, from the means already detailed. It is necessary, however, in order to secure permanency in the cure, that the means be employed for some time after the patient is apparently recovered.

When the ulceration of cartilage has made any considerable progress before the cure is effected, the joint is generally anchylozed by the remedial process. Cartilage has not been observed by our author to be ever regenerated, when once abraded.

CHAP. V. *On a scrofulous Disease of the Joints, having its Origin in the Cancellous Structure of the Bones.*

Mr. Brodie conceives that, although scrofulous persons are more liable than others to the preceding diseases, yet that there is no necessary connection between scrofula and them. But there is another malady which affects the joints, having all the characters of scrofula, and which is usually accompanied by general scrofulous symptoms. In this disease, the cancellous structure of the bones is the part primarily affected; in consequence of which, ulceration takes place in the cartilages covering their articulating surfaces. When this obtains, the subsequent progress of the disease is, in many respects, the same as where this ulceration takes place in the first instance. This affection attacks only those bones, or portions of bones, which have a spongy texture, as the extremities of the cylindrical bones, and the bones of the carpus and tarsus, affecting ultimately the joints. It occurs most frequently in children, and is rare after thirty years of age —the hip and shoulder are the joints less liable to it than the others. While the disease is going on in the cancellous structure of the bones, before it has extended to the other textures, and while there is still no evident swelling, the patient experiences some degree of pain, which, however, is never so severe as to occasion serious distress. After a few weeks or months, the parts external to the joint begin to sympathise with those within it; and serum and coagulable lymph being effused into the cellular membrane, the joint appears swollen. The swelling is puffy and elastic. Lameness and pain are now observable. As the cartilages continue to ulcerate, the pain becomes somewhat aggravated; but is not severe until abscess has formed, and the parts over the abscess have become distended and inflamed. The skin, under these circumstances, assumes a dark red, or purple colour. The abscess is slow in its progress; and when it bursts, or is opened, it discharges a thin pus, with portions of curdy substance floating in it. A succession of abscesses generally takes place, some of them healing, while others remain open in the form of fistulous sinuses; at the bottom of which, carious bone may be felt by the probe. The patient may go on a long time in this state, the constitution unaffected by the disease; but in less fortunate cases, the patient becomes subject to hectic fever, under which he gradually sinks, unless the cause be removed by amputation.

*Treatment.* In attempting the cure of the scrofulous disease of the joints, it is necessary to bear in mind the morbid condition of the general system; and consequently, that general remedies are of still greater necessity than local ones. Mr. Brodie has seldom seen advantage from local abstractions of blood, blisters, liniments, or caustic issues. Cold evaporating lotions seem to check, in some degree, the extension of the disease from the bones to the other parts, and to retard the occurrence of suppuration, and they may be employed with advantage, in the early stage of the complaint. Nothing, however, is of such importance as perfect quietude. All motion, and pressure of the articulating surfaces against each other, is likely to promote the ulceration of the cartilages, and hasten the formation of abscess. We cannot indeed expect that quietude will amend the scrofulous state of the bones themselves; but it may do much towards preventing the disease from affecting the other parts.

During the formation of abscesses, fomentations and poultices may be employed, to hasten their progress and relieve pain. When the abscesses have ceased to discharge, and there does not appear any farther disposition to suppuration, then we may expect that a curative process, by means of anchylosis, is about to commence. At this period, pressure by stripes of linen, spread with soap cerate, or other adhesive plaster may be usefully applied circularly round the limb. This will promote the healing of the sinuses, and favour the union of the ulcerated bony surfaces.

In respect to constitutional treatment, the sea coast and mild nutrient diet, with chalybeates, are the best. If the steel medicines excite any febrile movement in the system, the mineral acids may be substituted. Mercurial purgatives must be occasionally used. When the organization is completely destroyed, and the constitution is suffering from the local affection, then amputation is the only resource.

Mr. Brodie here adverts to the frequency of scrofula developing itself on some of the interior viscera when the articular disease is removed; a circumstance which, though not sufficient to warrant the surgeon in forbidding an operation altogether, in all cases where it is not actually and indispensibly necessary to save the patient's life, yet is quite enough to make him cautious not to recommend and urge the operation too strongly.

### CHAP. VI. *Caries of the Spine.*

This disease may take its origin sometimes in an ulceration of the intervertebral cartilages, and at other times in a morbid condition of the cancellous structure of the bodies of the vertebræ. The two diseases, however, cannot be distinguished during life.

Two orders of symptoms may arise from spinal caries; those which are consequent on the morbid condition of the vertebræ themselves; and those consequent on pressure or irritation of the spinal marrow. Our author thinks that when the disease is situated above the lumbar region, these two sets of symptoms are combined; whereas, when the vertebræ of the loins are alone affected, the latter set of symptoms are generally wanting.

Caries of the lumbar vertebræ usually occasions a pain in the loins, followed, after a longer or shorter period, by external abscess, shewing itself in the groin, or in some other situation, constituting one of the diseases which are confounded with each other under the name of psoas or lumbar abscess. The symptoms produced by those other cases, where caries is followed by curvature of the spine, and affection of the spinal marrow, are so well described by Pott, in particular, that Mr. Brodie has thought it unnecessary to enter upon them.

*Treatment.* For the reasons abovementioned, our author is very brief on this head too. Quietude, the horizontal position, and issues, are the means recommended by Mr. Brodie, while he acknowledges, at the same time, that some cases occur, "in which the caustic issues seem to be productive of little or no benefit." P. 290.

The last Chapter is on Inflammation of the Bursæ Mucosæ, in which leeches and blisters are recommended in the early stage, while frictions are employed in the ulterior periods for the purpose of promoting the absorption of the fluid. When this last cannot be effected, Mr. Brodie thinks the tumour may be punctured with safety.

We have now given such an analysis of Mr. Brodie's volume as precludes the necessity of any concluding observations. The work evinces throughout, superior talent, great modesty, and much discriminative acumen. To say less would be unjust—to say more would be unnecessary.

### Puerperalogy.

### VII.

*Facts and Observations relative to the Fever commonly called Puerperal.* By JOHN ARMSTRONG, M. D. Physician to the Fever Institution of London. Octavo, pp. 240. Second Edition, London, 1819.

AMONG every people on the face of the Earth, barbarous or refined, the parturient state of woman, doomed by the fiat of her Creator to "bring forth in pain," excites the sympathy and commiseration of all around her. As civilization and luxury advanced—and as new diseases were engendered, or morbid predispositions engrafted on the human constitution, the pains and penalties of parturition were fearfully multiplied. In the present state of society, in these countries at least, danger waits on every stage of labour; and when the whole process is happily terminated, the experienced practitioner well knows that a fever, little less fatal than the plague itself, is liable every moment to be kindled up, and quickly convert the mansion of joy into the house of mourning.

An attempt to elucidate the nature and treatment of this dangerous attendant on the puerperal state, appears to have been one of the earliest essays of Dr. Armstrong, a physician who, in riper years, has earned, and deservedly earned, the highest honours which his contemporaries can bestow — REPUTATION. Although we are not without some experience in the disease under review, yet our principal object shall be to set a clear and copious analysis of Dr. Armstrong's work before our readers, convinced that the pages of this Journal cannot be better freighted than with matter flowing from the pen of so judicious and accurate an observer as our author has long proved himself to be. We wish it then to be understood that, in the following analysis, we are always speaking the sentiments of Dr. Armstrong, though often in our own language and idiom, unless where we unequivocally come forward with our own personal remarks, or introduce the opinions of other practitioners.

Considering that much of the discrepancy of opinion respecting puerperal fever may arise from the confounding the disease with others which simulate its characters, Dr. Armstrong very properly, we think, sets out with a section on

I. DIAGNOSIS. The first sentence in this division of the work, involves a question of serious solution, and is delivered with an air of confidence and authority which will require all the weight of our author's name to render it perfectly convincing.

"If, in a therapeutic view, as shall be afterwards shewn, the puerperal epidemic should be thought essentially different from the ordinary peritonitis of lying-in women, the distinction will, most assuredly, be made at the hazard of life." P. 13.

In a subsequent Section, Dr. Armstrong maintains with great ability, force, and ingenuity, not indeed the perfect *identity* of the sporadic and epidemic forms of the disease, but their similarity of character in what may be termed their internal pathology; and of this we shall have an opportunity of speaking presently. In the mean time he draws the attention of his readers to the distinctions between puerperal peritonitis and milk fever, after-pains, inflammation of the uterus, and that ephemera called the "weed."

1. In the milk fever there is throbbing, irritation, and enlargement of the mammæ; while in puerperal, the pain begins and continues in the abdomen, the breasts being, for the most part, more flaccid than usual.

2. In after-pains, pressure can be borne, *at intervals*, without uneasiness — not so in puerpera. With the first, there is *not* fever; with the second there is an accession of fever, and an uncommon rapidity of pulse. Simple hysteritis is known by a burning, throbbing pain, fullness and weight in the uterine region—by urgency to make water, which is passed with pain and difficulty—by the uterus itself feeling hard, hot, and exquisitely sensible on pressure—by violent pains darting to the groins, thighs, and back,—and by increased uneasiness in the erect posture. When to the above symptoms are added, thirst, heat of skin, quick pulse, nausea, and suppression of the lochia, there can be little question of uteritis.

Nevertheless, as our author truly observes, when we reflect that the uterus after delivery is, as it were, a recently wounded organ, we cannot wonder that hysteritis very often constitutes a part of the abdominal inflammation attendant upon puerperal fever, whatever may be told or taught to the contrary. The experience of our author confirms that of Dr. Denman, that in no instance has a woman been attacked with puerperal fever, who had an abscess in the breast.

3. The ephemera or weed is ushered in by strong rigors, followed in less than an hour by heat, thirst, and general excitement. The whole train of symptoms being terminated in twenty-four or thirty hours by profuse perspiration. Here too is the absence of abdominal irritation.

Having thus sketched out what is *not* puerperal fever, we come to what Dr. Armstrong thinks is the disease.

"The puerperal fever, especially under an epidemic character, sometimes creeps on in a very insidious manner, the abdominal inflammation being marked by an oppressive languor, and a diminished sensibility of the nervous system; yet, in such cases, the disease may generally be detected by the great frequency of the pulse, by the quickened respiration, by flatulence of the stomach, and by the patients shrinking when pressure is applied over the abdomen, though they previously made little or no complaint of pain in that part. In those cases of puerperal fever which commence with uneasiness in the head, the patients sometimes complain of little or no pain in the belly for two or three days after the first attack; but the breathing is always quicker, the pulse more frequent, the abdomen rounder and fuller, and the stomach more flatulent than natural. Forcible pressure on the belly is one of the best tests of the abdominal inflammation; and when it is made, the practitioner should always watch the countenance of the patient, for that will undergo an immediate change if the pressure give uneasiness. But even if pressure on the belly should induce no change of the countenance, still, if the pulse should be rapid, and the respiration anxious, it is safest to treat the case as if abdominal inflammation openly existed. Sometimes, in the puerperal fever, as in typhus, there is an universal soreness of the flesh, a condition of the surface which may be extremely embarrassing; for if the practitioner should, at first, press his hand over the abdomen, the patient would shrink much; but if he tried pressure on other parts, she would still do the same, so as to leave doubts as to the presence of visceral inflammation. In such cases, however, the brain and spinal cord are commonly more or less inflamed, of which the soreness of the surface is symptomatic; but if the patient lie upon her back, with the feet drawn upwards, and if stretching them suddenly give pain, and if the breathing be hurried, and the stomach flatulent and irritable, abdominal inflammation may be presumed also to exist. Thus it will appear, that two opposite states of the nervous system may occur in the puerperal fever, one in which the sensibility is diminished, and another in which it is increased; and without due care on the part of the medical examiner, both may contribute to mislead him as to the actual state of the abdominal viscera." P. 24-5.

Dr. Armstrong conceives, and with reason, we think, that dark, slimy, and fœtid stools, "somewhat like thin, dirty yellow paint, thrown out in considerable quantity in the course of the disease," are highly characteristic of puerperal fever; to which we may add, from our own experience, that the patient's bed generally emits a peculiar unpleasant odour, resembling cabbage-water. The indifference of mothers about their offspring, or surrounding objects, is also thought by our author and others, to be peculiar to this disease. The countenance manifests alarm and solicitude, as though the person affected was the subject of two different emotions at the same time. Nothing decisive can be learnt from the state of the lochia. In the greater number of cases, in the Sunderland epidemic, they were gradually diminished or suppressed, *after* the full development of the fever—in fact, the best authorities might be quoted to prove that the lochia are affected in some cases, but not in others, though they "are most frequently diminished or suppressed, in the progress of the puerperal fever."

Dr. Armstrong observes, that Dr. Hamilton, junior, in his lectures, maintains that the lochial discharge is *not* suppressed in true puerperal fever, and partly upon this assumption, attempts to distinguish it from other affections of child-bed resembling it. This distinction, Dr. A. thinks, must fall to the ground; "since, so far from being supported, it is directly opposed by a host of facts." Finally, abdominal pain or soreness; quick anxious breathing; unusual frequency of the pulse; increased temperatuie; anorexia; prostration of the vital and voluntary powers, with an unnatural condition of the excrements, are among the chief pathognomonic signs of puerperal fever.

Our author wisely exhorts practitioners of midwifery to visit their patients very frequently for several days after delivery, and narrowly to observe the state of the pulse, tongue, stomach, bowels, respiration, countenance, and skin, since it is in the provident ánticipation of disease that the medical man most strikingly displays the force of his understanding, and the efficacy of his art.

II. Prognosis. Although Dr. Armstrong so far coincides with the common opinion as to the great danger of puerperal fever, and is convinced that part of its fatality may be fairly ascribed to its natural tendency; yet he is fully persuaded, that it may generally be arrested in the beginning, and that "much of its fatality has been occasioned by our great caution, timidity, and indecision in treating it." Still a cautious prognosis is to be given, even in the most favourable cases.

It seems agreed upon, by all accurate observers, that the earlier the attack the greater is the danger, and that those whose powers of feeling are much diminished from the beginning, and who consequently complain but little, generally sink under the pressure of the disease.

"On the other hand, an excess of sensibility is always to be dreaded."

The slightest approach to mental confusion or delirium is an inauspicious sign, at any period of the complaint.

"An agitated countenance, with a hurried unconnected manner of speaking, constant sighing attended with a tossing of the arms, pain and oppression of the chest, visual deceptions, imaginary strange sounds and voices, muttering and stupor, are among the most unfavourable symptoms." P. 37.

The time which the disease has continued must greatly influence the prognosis, since irreparable injury is often sustained by some of the abdominal viscera in the course of twenty-four hours. Dr. John Clarke observes, that scarcely any fever, except the plague, destroys life so rapidly as the puerperal fever, and our author himself saw an instance where it proved fatal in forty-eight hours. At any time, Dr. A. correctly remarks, when the disease has existed more than twenty hours, rigors are highly alarming. After copious venesection in these cases, patients sometimes become cold, faint, and shivery, which symptoms, however, need not create alarm, provided the operation has been opportunely performed, for a gradual warmth and moisture of the surface will succeed, with reduction of the pulse. If, on the other hand, the bleeding was too late, or when the stage of collapse was about to commence, nothing can be a more dangerous symptom than the continuance of this coldness, shivering, and faintness.

An open state of the bowels immediately before delivery generally tends to mitigate the severity of an attack, and a diarrhœa coming on early, will often carry off the patient. If the respirations amount to fifty in the minute, being feeble, with an extremely weak, quick, and compressible pulse; if there be frequent coffee-coloured vomitings, an increase of abdominal distention, repeated shiverings, and a universally cold, damp skin, the case may be pronounced desperate.

"On the contrary, when the respiration grows easy, deep, and slow; when the pulse comes down, and ceases to be variable; when the stomach retains the food and medicine, the stools continue copious, the tension and pain of the belly abate; when the skin breaks out into a warm sweat, the tongue becomes clean and moist, and especially when the fresh discharges of the lochia, and secretion of the milk take place, the symptoms fully authorize a favourable opinion." P. 42.

III. Prevention. As costiveness often assists in the production of puerperal fever, so the timely exhibition of mild purgative medicines, such as castor oil, may be reckoned one of the best preventives. One copious evacuation should be procured daily during pregnancy. The patient should avoid getting out of bed to have her linen changed, as she is then liable to a chill, as well as hæmorrhage. During labour, the os uteri should not be irritated by frequent and unnecessary examinations; neither ought the placenta to be extracted too hastily. Mr. Ferguson, an experienced practitioner in midwifery, of Bishop Wearmouth, has hardly ever seen puerperal fever succeed uterine effusions merely arising from defect of contractility in the uterus; but has often seen it follow those hæmorrhages arising from an injury sustained by that organ. This, our author thinks, is an important fact, and perhaps may, in part, tend to explain some of the existing discrepancies of authors, some of whom assert that floodings occasion, and others that they prevent the disease. In all cases, after profuse floodings, the prostrate powers of the system should be slowly and cautiously raised, thereby to avoid any secondary affections of excitement. It is almost needless to say, that the lightest regimen is proper for many days after delivery.

IV. Pathological Remarks. Dr. Armstrong has wisely determined not to connect hypotheses with the plain evidences of symptoms and dissections; by which, in his opinion, a true knowledge of pathology can alone be established.

"If an unprejudiced practitioner were called to a woman shortly after parturition, and found her labouring under an oppressive fever, the abdomen painful and distended; the skin hot; the tongue dry; the pulse very quick; the breathing hurried, and the secretions probably diminished or suppressed: and further, if he finally saw his patient fall a victim to the complaint, and, on accurately dissecting the body afterwards, discovered the most extensive traces of an abdominal inflammation, without any other appearances sufficient to account for death, he would at once conclude, that the disease was of an actively inflammatory nature, and would determine for the future to treat it, and every similar affection, with the greatest promptitude and decision. Such a conclusion and determination I would most earnestly recommend every medical man to form; first, because there is perhaps no disease more uniform than puerperal fever, in the symptoms and morbid derangements which it induces; and secondly, because it can only be combated with the probability of success by antiphlogistic means." P. 63.

In answer to those who contend that the low child-bed fever is specifically distinct from puerperal peritonitis, Dr. Armstrong asserts, that all the anatomical examinations which have been made in the low child-bed fever incontestibly prove, that "if there be any difference between it and the puerperal peritonitis, with regard to their inflammatory disposition, that difference merely consists in degree, the vestiges of inflammation being more strikingly evident, and extensively destructive in the *former* than in the *latter*." For the truth of these observations, he refers to the writings of Denman, Leake, Home, Hulme, Clarke, and Gordon, and with these his own post mortem researches agree.

"From all that has been advanced, then, it may be laid down as a general proposition, that abdominal inflammation, directly or indirectly, is the cause of the fatal termination of all the varieties of the puerperal fever. But though I have strenuously contended, that the puerperal peritonitis, and the low child-bed fever may be pathologically considered as modifications of the same disease, yet I have avoided the term identity, because I have no inclination to enter into abstract discussions or nominal disputes. Nor will I take upon me to assert, that there is always a perfect sameness, since there necessarily must be such a difference as arises from the peculiarities of the patients, from the parts within the abdomen most decidedly attacked, and from the influence of the seasons and other circumstances. Nay, I have no objection to grant that the inflammatory character of this disease sometimes conceals, and even appears to lose itself in an almost unequalled prostration of the powers of the system. What I wish, however, particularly to insist upon is this, that the low child-bed fever, and the puerperal peritonitis, are so far the same as to require the depletory practice; only in the *former*, this practice must be more promptly and powerfully applied, as the time in which the professional man can be useful is much shorter, on account of its greater intensity." 72.

In the Sunderland Epidemic, many cases, Dr. A. avers, bore the character of a simple inflammation of the peritoneum; while others again, assumed a more mixed and malignant character, from the greater depression of the general powers. Our author, in his first edition, strenuously maintained that the disease sprang from contagion; but he now wavers, or at least modifies his creed.

"An attentive perusal of various Treatises, and a cautious survey of those cases I have myself seen, would authorize me to conclude, that the puerperal fever may proceed from a variety of causes; and future observation will probably bear me out in affirmings, that any circumstance which gives a general shock to the system, is fitted to produce the disease in habits already predisposed." P. 74.

V. Treatment. Whoever glances over the works of those who have written on puerperal fever, will be astonished at the variety and discrepancy of opinions which have prevailed on the subject of its treatment.*

Dr. Armstrong shrewdly conjectures, that much of this diversity proceeds from want of a proper regard to the distinctions between the stage of excitement and the stage of collapse. On this account, we shall endeavour to convey a full idea of our author's delineations and distinctions between these stages, as we agree with him on the great importance of accurate discrimination in this part of the subject.

In all cases, puerperal fever is ushered in with some degree of diminution in the force of the pulse, and also in the animal temperature, an invariable prelude to the establishment of the true febrile state. This is to be accounted the first stage; but as it is a very obscure one, and common to other affections, the character of the puerperal fever is rarely declared unequivocally till the reaction

* See page 645 of Burns' Principles of Midwifery, where he enumerates the clashing practices of Denman, Leak, Gordon, Butter, Manning, Walsh, Hulm, Doulcet, Whyte, Clark, Kirkland, Hull, Hamilton, &c.

exist. The occurrence of that reaction, and its continuance to its highest point, our author denominates the stage of excitement; "and on the other hand, the marked declination of that excitement, and the symptoms of constitutional exhaustion which supervene, shall be denominated the stage of collapse." P. 77.

As soon as the heat becomes higher, and the pulse quicker than natural, the stage of excitement may be considered as established.

"In this stage, the skin is commonly dry, as well as hot; but, in some instances, it is partially damp while it is universally hot, and this is particularly liable to happen where the pain is violent, or where the stomach is affected with nausea or vomiting. The pulse is hardly ever less than 120 during this stage; and, in some rare cases, as high as 140, or even higher. In general, the blood does not flow in a soft, easy, tranquil current, but comes against the finger with a vibratory sort of motion; and more than ordinary pressure is commonly requisite to stop its course along the artery, which, in such cases, feels hard and tense, like a cord upon the stretch. Yet there are some instances in which the pulse is very quick, and peculiarly soft and compressible from the first occurrence of the stage of excitement; though this, so far from being more favourable, is considerably more dangerous than the hard resisting pulse, for it marks a relaxation of habit highly to be dreaded in every form of fever. In this stage, too, the patient complains most of the abdominal pain and soreness, breathes above thirty times in the minute, and rather anxiously; has a white, foulish tongue, considerable thirst, and much febrile restlessness and irritation. The belly is generally bound, and bile or mucus vomited in some cases; but in others, the stomach is little disturbed, though its powers in most are prostrate." P. 78.

Such is Dr. Armstrong's excellent symptomatology of the stage of *excitement*. That of *collapse* we shall condense in our own language and style.

In this stage the pulse becomes exceedingly weak and small, ranging from 110 to 140 in the minute. The respiration becomes much quicker, not uncommonly 50 per minute; the heat now begins to decline, and is not equally diffused, as in the excitement. Cold partial perspirations first break out about the face, neck, and extremities; the central parts of the body often remaining dry, and of a superficial glow, for some time afterwards. Vomiting of food and medicine now is common, occasionally tinged with a dark grumous matter, the belly being loose, and the abdomen distended with flatus. The tongue is variable—brown, black, or red, rough and parched. The thirst is unquenchable; the patient is less capable of assisting herself; the countenance is sunk, and often agitated; the face and lips cadaverously pale; the patient lies prostrate on her back, often moving her hands and drawing up her feet, or tossing about her arms, with moaning or muttering; and, as the final close draws near, relaxed, damp, and cold skin. In fine, the stage of excitement is marked by inflammatory, and the stage of collapse by low malignant symptoms, the degree of the latter being almost invariably in proportion to the degree of the former.

It is true that no one of the above symptoms, taken singly, can be depended on as distinctive between the stage of excitement and that of collapse; yet, as several of them accompany or succeed each other, they may together enable the practitioner to discriminate each of these states in the puerperal fever.

The stage of excitement is variable as to duration, sometimes terminating in twenty hours, at others extending to seventy, even when the inflammation is of the acute kind. The period of collapse is also uncertain. If accompanied by gangrene of any of the viscera, a few hours terminate the scene; if by suppuration, it is generally mortal in a few days. But where it is accompanied, as generally happens, with a serous effusion into the abdomen, the disease may run out longer; and, in some rare instances, terminate in recovery.

If Dr. Armstrong may be allowed to judge from his own experience, he would lay it down as a general rule, that the abdominal inflammation is the greatest in those cases which are attended, from the beginning, by most oppression of strength, and of the vital powers; hence, it will be found a fatal delusion to be deterred from early bleeding and purging by the semblance of debility, which only serves as a covering to obscure the destructive progress of the abdominal inflammation. The puerperal fever, under every form, will only be found remediable in the stage of excitement; at least, nothing can be done with the probability of success when the general collapse supervenes.

"In the first obscure stage, in which the puerperal fever is ushered in by paleness of the surface and oppression, or by a pretty distinct attack of rigor, the animal heat is almost always below the natural standard; and it is of great consequence towards lessening the degree of the subsequent excitement, that the natural temperature of the surface should be as rapidly restored as possible. When a tepid bath, therefore, can be speedily obtained, it frequently has an excellent effect, if it can be used without fatiguing the patient; and where that cannot be speedily had, the blandest tepid drinks must be recommended, and bottles of warm water put to the feet and stomach, which are good substitutes for restoring the circulation of the surface, and relieving the internal organs from an over pressure of blood. An enema should be first ordered, and a dose of castor oil afterwards, by way of more effectually clearing the bowels. All kinds of diffusible stimuli ought to be expressly prohibited at this period, for they are extremely pernicious. The animal heat having been restored, and the character of the disease developed, not an instant of time ought to be lost in attempting to arrest it: and even in those cases where some doubt may exist respecting the actual presence of inflammation, it is better, as a general rule, to bleed at once; for by trusting to a purgative, or to some other secondary measure, the patient may thereby be lost from the delay; and even in ordinary fever occurring in child-bed, no harm can result from early and moderate venesection.

"In acute diseases, it has been too much the practice to confide in one principal measure; but in general, they may be best removed by a series of antiphlogistic measures. Although strenuously insisting upon the utility of venesection at an early period in the stage of excitement, yet it was never my intention to affirm, that it is, of itself, generally equal to the removal of the puerperal fever. On the contrary, it has generally failed in my hands, unless followed by free purgation; and on inquiry, too, I find, that the patients of those authors who adopted venesection generally died, when the purgatives did not act at all, or only imperfectly. It is not then, simply bleeding and purging in which I have so much confidence, but in copious bleeding, immediately succeeded by copious purging, or rather in the powers of these two means simultaneously exerted on the disease at the onset." P. 117-18.

As there is often a deceitful remission about the second or third day, laxatives should rarely be intermitted till after this last period. The gastric irritability is generally lessened by venesection; immediately after which, a large cathartic enema should be administered, as evacuating the lower part of the intestines is favourable towards the retention of the purgatives afterwards exhibited. In such cases, calomel suspended in mucilage, is more likely to be retained than any other, especially if combined with a little opium, which does not tend to constipate in inflammatory affections of the abdomen, as Dr. A. has witnessed in numerous instances, and as we ourselves have almost uniformly found to be the case. If the sickness continue after the bowels are freely opened, it is a sign of some visceral mischief, demanding the prompt use of the lancet. Warm fomentations to the abdomen, though secondary means, are very useful auxiliaries. Dr. Armstrong cannot speak of cold applications to the belly, as recommended by Dr. Sutton, not having ever ventured on them. We were a few days ago informed by a medical friend, that Dr. Temple, of this metropolis, has been in the habit, for very many years, of treating all cases of peritoneal inflammation, especially that form called enteritis, by cold evaporating lotions over the surface of the abdomen, employing, of course, the other usual means, and with the most decided success. The friend who communicated this information, a man of great talent and observation, has also for some years followed the same course, and speaks of the measure in terms of the highest praise.

Dr. Armstrong justly remarks, that no fixed rule, as to the quantity of blood that ought to be abstracted in puerperal fever, can be laid down. We must be guided by the effects produced. We ourselves make a point to bleed till the patient unequivocally admits that the pain is gone, or until she faints, which will generally produce the same effect. On this account, we always draw blood from the patient while she is in the supine posture in bed, and that from a pretty large orifice, but not from such an immense one as leads quickly to syncope, before any material quantity can be abstracted. During the venesection, we keep the finger constantly on the radial artery, and the eye steadily fixed on the patient's countenance. When the abstraction draws towards the point of utility, we generally feel the pulse soften, and fall a little in frequency, while the blanching countenance gradually loses the peculiar character of suffering which attaches to this and most other abdominal inflammations of the serous tissues.

Within these few weeks, we demonstrated to three medical gentlemen who were present, the efficacy of decisive and bold practice in this most dangerous malady. A puny and delicate woman, about 33 years of age, had suffered, a month before delivery, a smart attack of pulmonic inflammation, requiring two bleedings, together with the other auxiliary means to subdue it. The waters broke some days before labour commenced, and the labour itself was tedious, being an arm presentation, followed by the head, and terminating favourably without artificial aid. During the last few hours of labour there certainly was tenderness of the abdomen, on pressure about the umbilical region, and Mr. Alcock, an able practitioner of this city, prognosticated puerperal peritonitis; and even then considered that venesection might be advisable, before the expulsion of the fœtus. This tenderness, however, went off entirely after delivery, and the woman continued remarkably well, with lochial discharge and lacteal secretion, for thirty-six hours; at which time she was seized with pain and tension of the abdomen, strong fever, and in short, all the symptoms of puerperal peritonitis. As the attack commenced at eleven o'clock at night, we did not see her till nine the next morning, when we immediately abstracted sixteen ounces of blood, which caused faintness and abatement of the pain Ten grains of calomel were administered, and in an hour, a mixture of infusion of senna, sulphas magnes. and tinct. jalapæ, which was ordered to be repeated every two hours, till the bowels were completely evacuated. The abdomen to be frequently fomented. During this day she passed ten motions, which filled a large pot de chambre with excrementitious matters, not very unlike what Dr. Armstrong has described above. At nine in the evening we visited her, when Dr. Prevost, of Switzerland, Mr. Birckhead, of Baltimore, and Mr. Wilmore, of Worcester, were present. Notwithstanding the bleeding, and the profuse purging, neither the general fever nor the local inflammation was subdued. The abdomen was somewhat less tense, and the greatest pain and tenderness were complained of over the region of the womb. The woman was weak, the respiration quick, and the countenance bad. The disease had now ravaged for twenty-two hours, the lochia were greatly diminished, and the lacteal secretion arrested. As we were convinced that the stage of excitement would not last long in such a delicate subject as we had now to treat, we acted with firmness and decision. In the presence of the above-mentioned gentlemen, a vein was opened as the patient lay in bed, and while we watched the countenance, and kept our finger on the artery, we abstracted blood till the face became completely exsanguious, and till every sensation of abdominal pain was extinguished. This required twenty-eight ounces. A large blister was applied over the abdomen, and although the catharsis was going on in full force, we exhibited a dose of calomel, pulv. antimon. and extract of hyoscyamus. During the whole night, one continued purging was kept up, and all the next day. The pain returned no more, the fever vanished, and she had a rapid convalescence and perfect recovery.

Now, if a puny, delicate, and weakly inhabitant of London, reduced by a series of untoward illnesses and accidents, could bear up against, and derive advantage from such depletion as this, can we calmly give ear to those tales of imaginary debility, which are so boldly advanced as reasons for abstaining from active measures in the excitive stage of puerperal fever, by men grown grey in error, and blind to the light of truth?

Dr. Armstrong here relates several very interesting cases of hospital puerperal fever, which he had an opportunity of attending in a public Institution in London, and where the practice was equally efficacious as in private life.

"In confirmation, too, of this opinion, Dr. Gooch, whose experience in the hospital puerperal fever has been so extensive, recently told me, that though he had seen more formidable cases in hospital than in private practice, yet even in the former he had lost very few. From all he has seen of the disease, he has been led to the conclusion, that the only effectual remedies are bleeding and purging very boldly, and very early employed." 151.

Dr. Ramsbotham also has long and advantageously employed bleeding and purging in the various forms of puerperal fever, as presented to his observation in this metropolis. In respect to the choice of purgatives in this fever, much discrepancy of opinion prevails—some preferring calomel and the more drastic substances, while others give the preference to castor oil. Dr. Armstrong has observed that the East India castor oil is very pale, and merely laxative; while the West Indian, which is of a dark colour, is strictly a purgative. This distinction, therefore, should be borne in mind. When good, Dr. Armstrong has generally found that this oil occasions as copious stools as almost any other aperient, and with much less irritation than most. These two circumstances, therefore, render it a valuable medicine in many cases. But in puerperal fever, we shall generally find, that one or two full doses of calomel at the beginning, will be the best plan, keeping the bowels open afterwards with castor oil, or salts and senna, as may be deemed most advisable. In respect to large doses of calomel, Dr. Armstrong appears to waver a little in his sentiments, and leaves the subject open to future experience. For our own parts, while we believe that it is only upon extraordinary emergencies that they are necessary, we are at the same time perfectly convinced of their safety and innocence. Even Dr. Armstrong has fallen into an error, in believing that scruple doses of calomel to people in health, would be productive of mischief. Mr. Cunningham, a medical gentleman of great talents, tried the experiment on himself, in health, and completely disproved the above preconception. He has also been in the habit, for some years, of curing primary symptoms of syphilis, by scruple doses of calomel, exhibited twice or thrice a day, till the mouth became sore, when the ulcers almost immediately healed, and returned no more. Mr. Boyle also, in a late number of this Journal, has published some cases where syphilis was treated by calomel in scruple doses, with rapid effects in eradicating the disease. In all these cases, the medicine was exhibited to men, otherwise in perfect health, and yet without any injurious consequences, or indeed without any extraordinary effects at all. In the Bristol Infirmary, Mr. Bedingfield informs us, that calomel, in scruple doses, is given with success in chronic rheumatism, and of course, where there is no constitutional commotion. Finally, we have, in numerous cases, exhibited the remedy to people in *general* health, without any inconvenience; and therefore, we think that there is the most decisive evidence of its harmless qualities, while the opinion of its injurious effects, in health, rests on preconception alone. We indeed agree with Dr. Armstrong, that remedies, *in general*, act very differently on the constitution in health and in disease; for instance, opium: but, to the calomel, in the doses above mentioned, the rule does not properly apply, since the *a priori* arguments on its effects in health fall before the evidence of facts.

Our author here takes up the same ground with Dr. Porter, in our last number, respecting the change which calomel effects on the colour of the stools. Our own attention has been a good deal directed to this very subject, and we cannot entirely coincide with these two authors, highly as we respect the talents of both. We have five children of our own, and have repeatedly given calomel in various doses, but generally small ones, to these, both in health and disease, watching narrowly the effects. In *health*, we found that very little alteration was made in the stools, except that of rendering them somewhat more copious, and more yellow. But where the calomel *griped* much, then *green* and mucous matters were brought down. In febrile and abdominal derangements indeed, the calomel very frequently causes, or we would rather say *dislodges*, green and fœtid secretions from the cells of the colon and the valvulæ conniventes. We are inclined to the opinion that the medicine *dislodges* rather than creates these coloured evacuations, from the circumstance of the fœtor which we have almost invariably found accompany them, and which we have strong reason to believe arises from their delay in the intestinal canal. We would recommend our younger brethren of the profession to acquire a habit of recognizing the morbid *effluvia* from alvine excretions, as of much more consequence than the *colour* of the evacuations, and from which much more is to be learnt.

Dr. Armstrong's remarks on the operation of opium are much more original and interesting than those on calomel. Having imbibed the common prejudice against this remedy in visceral inflammations, he has only arrived at the knowledge of its true operation by very gradual steps.

"Opium, when given in health, constipates the bowels; but this is so far from being the case in gastritis and enteritis, that it tends to assist the action of purgatives; and when exhibited in those complaints, in conjunction with proper depletion, it may fairly be accounted one of the best remedies." 171.

In answer to the objection that the good effects of opium resulted from its combination with calomel, Dr. A. states, that in some urgent cases he has been compelled to trust to opium merely, when previous bleeding and purging had produced little or no alleviation of the symptoms; and in these cases the efficacy of opium was so remarkably great, as to embolden and justify him in giving it in similar cases, when the ordinary means had failed.

In the Fever Institution of London, it has sometimes happened, that convalescents from protracted fevers have been seized with enteritis or gastritis, where blood-letting was inadmissible. Here early and full doses of opium, in conjunction with blisters and the mildest laxatives, have afforded the most perfect relief. The two most remarkable effects of opium in these diseases, were the relief of the pain and the reduction of the pulse, followed generally by sleep.

"Pain, it is well known, is a direct irritant to the heart, and thence to the arteries; and in many cases, thus prolongs the inflammation."

"In peritonitis, strictly so called, where the pain and tenderness are diffused, it is by no means so useful, its benefit being most conspicuous where the pain and tenderness are *circumscribed*, as in enteritis, gastritis, or hysteritis."

Even in puerperal fever, Dr. Armstrong thinks that opium may be given with considerable advantage, where the local pain and constitutional irritation are excessive, though bleeding and purging must, of course, be premised or conjoined. In the stage of collapse too, where bleeding is highly prejudicial, opium may perhaps be accounted the primary measure, since the allaying of irritation is then the principal object. Whenever opium is administered in any species of abdominal inflammation, the dose should be large, since a small one often stimulates, whereas a large one is a direct sedative. The following sentence we have frequently illustrated in this Journal and elsewhere, and we are happy in the coincidence of Dr. Armstrong.

"Between that state of the nervous system called irritation, and that state of the vascular system called inflammation, there is a most intimate relation; so that in many instances, when we promptly allay irritation, we prevent inflammation, which would have otherwise occurred." P. 175.*

Even when inflammation exists with much pain, opium, by allaying the latter, often removes the former. We may here make observation, that Dr. Armstrong, in the *didactic* part of the volume, lays too little stress on local blood-letting, especially leeching the abdomen; and were it not that we see the *practice* in several of the cases, we should conclude, that he attached a very secondary importance to the remedy. We conceive, however, that where circumstances will permit, a dozen or two of leeches should very generally precede the application of the blister to the surface of the belly.

Dr. Armstrong concludes the body of his work with some observations on a peculiar complaint of child-bed, bearing a considerable analogy to that form of typhus fever which he has termed congestive. It is ushered in by sensations of chilliness, or by paleness or oppression without these sensations. In both cases, the vital powers are so prostrated, that no regular re-action takes place, as in common fevers; so that the surface remains cool throughout, or there are merely short, partial, and irregular flushes of heat. This shock in general suspends all the secretions, and the patient sinks with rapidity. In such cases, dissection does not reveal the usual remains of inflammation, the only morbid appearances being an unusual accumulation of blood in some part of the venous system, without any of those vermillion tints of the capillary arteries which denote the previous existence of inflammation. The disease is fortunately very rare, for it is very fatal. The treatment must consist chiefly in creating an artificial re-action—at least, by restoring the temperature of the surface, to enable the heart to keep up the balance between the arterial and venous systems. The warm bath, diffusible stimuli, &c. to bring on re-action; and then we are

* See Vialie on the Circulation and Excitement, in No. I. of this Journal.

to regulate or modify this re-action, according to its intensity.

We have so far o'erstepped our limits, that we are unable, at present, to notice several interesting communications in the Appendices, from Dr. Clarke of Dublin, and Messrs. Alcock, Wolfe, Gregory, and Gregson. The length of the analysis and the full exposition which we have given of the work, render it almost unnecessary to say a word on its general merits. The book is so much better than any other on the same subject, that it can only be compared with itself, or rather with its brotherhood.—Were we disposed to institute a parallel between this last production, and the two former on typhus and scarlatina, from the same pen, we should be inclined to apply to them the three degrees of comparison which we learnt from our Grammar some thirty years ago. We would call the work on puerperal fever *good*—very good—that on scarlatina, &c. *better*—but the work on typhus *best* of all. Indeed, when a physician has written such a work as that of Dr. Armstrong's on typhus, he labours under peculiar disadvantages in all subsequent enterprizes—disadvantages which Homer and Milton experienced in their turns.

In the work just reviewed, we have thought—but perhaps it was only fancy—that we could perceive a kind of rein kept by our author, on that bold, original, and independent train of *reasoning* which pervades the work on typhus. If this be the case, we are sorry for it.—Dr. Armstrong has no occasion to sacrifice an atom of his own conviction at the shrine of prejudice. His *reasonings* have hitherto reflected a lustre—and consequently an *illustration* over his *facts*; and it will be a great pity if he be deterred, by the cavils or sneers of envy or dullness, from that noble line of conduct which he originally marked out for himself. Against error and prejudice, and all who are enlisted under their banners, it is the duty of such writers as Dr. Armstrong to wage eternal war. Talent and steady independence of sentiment carry with them a magic force and power which no artificial barrier can ultimately restrain.

### CATALOGUE RAISONNÉE.

### I.

*Medical Botany ; or the History of the Plants in the Materia Medica, illustrative of the London, Edinburgh, and Dublin Pharmacopœias, arranged according to the Linnæn System.* No. I. pp. 12, with Six Plates. To be continued Monthly. London. 1819.

THE object of the Author is to give a condensed Botanical and Medical History of such plants as are at present used by the Medical Practitioner. We can very conscientiously recommend this useful publication to the Profession at large. The plates are elegantly executed, and beautifully coloured ; the letter-press on excellent paper, and the whole at the moderate price of three shillings and sixpence a Number.

### II.

*The Hospital Pupil's Guide ; being Oracular Communications addressed to Students of the Medical Profession, &c.* By ESCULAPIUS. Second Edition corrected. Octodecimo, pp. 96, with a Plate of Cheselden.

THERE are very few books of the same size that contain so much, and such good advice to the young student as this little volume of Esculapius. The only fault we have to find with him is, his recommendation of too many books to the pupil, and that without sufficient selection.

### III.

*Venus sine concubitu, vel*——By A. P. BUCHAN, M. D. Small Octavo, pp. 112. London, 1819.

THE title and composition of this performance are alike beneath the dignity of a man of science. It is a rivulet of reason winding through a wilderness of absurdity, calculated to excite the curiosity of the profligate—to augment the terrors of the hypochondriac, and to — draw patients to the author! It must have been some compunctious feeling that dictated the following sentence, for well might Dr. Buchan say—" That he has been, *in some measure*, deterred from submitting the following observations to the public, from a dread of being classed with those worst of empirics who, under the disguise of similar topics, fasten upon minds already infected by disease, and extort large sums of money, &c." By disseminating this work among the general mass of society, the author has done no good to the public, and no honour to the profession!

### IV.

*An Inquiry into the Effects produced on the Brain, Lungs, and other Viscera, and on the Nervous System, by Diseases of the Liver.* By THOMAS MILLS, M. D. Octavo, sewed. Pages 107. London, 1819.

IN this Pamphlet, Dr. Mills has dealt more in facts than in doctrines, and he has brought forward a large body of evidence to prove that a considerable proportion of disorders, *apparently* seated in the brain, lungs, and other viscera, as well as in the more extended nervous system, derive their origin from derangement of function or of structure in the liver, and consequently are to be cured by mercury, vascular depletion, and purgatives. Our readers are well aware, that Dr. Mills cannot lay much claim to novelty of opinion on those points; but he is entitled to the thanks of the profession for having recorded, and now made public a candid statement of facts in support of the abovementioned doctrines, accompanied by a train of very sensible and acute commentaries. As the pamphlet is published in a very cheap and unostentatious form, we can conscientiously recommend it to the serious perusal of every rank of the profession.

### V.

*Remarks on the Causes, Prevention, and Management of the present prevailing Epidemic Fever.* By W. O. PORTER, M. D. one of the Physicians to the Bristol Dispensary. Octavo, pp. 53, sewed. London, 1819.

THIS little Essay, though written in a somewhat popular style, is perfectly orthodox in its principles, enlightened in its views, and animated in its language. It is calculated to be very beneficial to both patient and practitioner, inasmuch as it enables the popular reader to distinguish the disease early, and, in that case, he is urged, by convincing arguments, to lose no time in availing himself of the Physician's aid. Dr. Porter traces the fever to aerial influence, and estimates its contagious power at a very low rate. His treatment is exceedingly judicious, and hinges on an accurate discrimination of the different stages of the disease, and constitution of the patient.

### VI.

*A Lecture on Dropsy.* By GEOEGE GREGORY, M. D. &c. &c. Octavo, sewed, pp. 40. London, 1819.

DR. GREGORY informs us that, so far back as 1810, he communicated to the Westminster Medical Society, some observations on dropsy, corresponding, in several points, with the views which Dr

Blackhall afterwards promulgated. He owns himself indeed, indebted, for many of these views, to Dr. Nevinson, of St. George's Hospital; a gentleman of whom we have lately heard so much, and from so many quarters, that we anticipate something from his pen, which may prove honourable to science, and useful to humanity. Dr. Gregory adopts the tenets and approves the practices of Abercrombie and Crampton. The latter gentleman's reports on dropsy will form a very long and very important article in our next number. Although Dr. Gregory's lecture is modestly addressed to the junior branches of the profession, we consider the doctrines inculcated (with the exception of a few theoretical views which we do not profess to understand) as deserving the attention of many heads which know themselves to be much older, and think themselves much wiser than Dr. George Gregory.

